# Biosurfactants and bioemulsifiers: An annotated selection of World Wide Web sites relevant to the topics in microbial biotechnology

**DOI:** 10.1111/1751-7915.12112

**Published:** 2014-02-14

**Authors:** Lawrence P Wackett

**Affiliations:** Department of Biochemistry, Molecular Biology and Biophysics, BioTechnology Institute, University of MinnesotaSt Paul, MN, 55108, USA

## Surfactant: Wikipedia

http://en.wikipedia.org/wiki/Surfactant

This page provides a primer on surfactants of all types, including biosurfactants.

## Methods for studying biosurfactants

http://www.ncbi.nlm.nih.gov/pubmed/20210700

Biosurfactants can be characterized using a variety of physical and spectroscopic methods, and this review discusses those methods.

## Biosurfactants book

http://www.landesbioscience.com/books/special/id/1791/

This page describes a comprehensive open-source e-book on biosurfactants. It was published in 2012, so it contains recent information.

## Biosurfactant research group

http://rkt.chem.ox.ac.uk/projects/biosurfactants.html

This academic research page provides a good overview of biosurfactants.

## Global biosurfactants market

http://www.prweb.com/releases/2012/11/prweb10158903.htm

While the full report is available only for a fee, this page hints at the importance of biosurfactants commercially.

## Biosurfactant-producing bacteria

http://www.ideaconnection.com/crowdfunding/isolation-and-characterization-of-biosurfactant-produ-00041.html

This page on the IdeaConnection website proposes research on bacterial biosurfactants.

## Biosurfactants and bioremediation

http://envismadrasuniv.org/Bioremediation/pdf/Biosurfactants%20and%20oil%20bioremediation.pdf

This page links to a comprehensive review article on the role of biosurfactants in bioremediation.

## Biosurfactant-mediated biodegradation of alkanes

http://www.amb-express.com/content/1/1/9/abstract

This is a typical example in which an alkane-degrading pseudomonad makes a rhamnolipid surfactant that aids in solubilizing and degrading the alkanes.

## Studies on novel surfactants

http://www.columbia.edu/cu/iucrc/research.shtml

This list of research at a university centre describes numerous projects on surfactants, including biosurfactants.

## Microbes and oil spills

http://www.dfo-mpo.gc.ca/science/publications/microbes/index-eng.html

This page contains a reprinting of a document from the American Academy of Microbiology that answers frequently asked questions about oil spills, largely from the perspective of how microorgansisms are impacted and impact the spill.

## Biosurfactant structures

http://www.biosurf.ugent.be/Biosurfactantia.htm

This introduction to biosurfacants contains text and shows chemical structures of characterized biosurfactants.

## Patents on bioemulsifiers and biosurfactants

http://nopr.niscair.res.in/bitstream/123456789/4804/1/JSIR%2065(2)%2091-115.pdf

This review arrticle contains information on more than 250 patents relevant to biosurfactants.

## Microbial production of biosurfactants

http://www.iisc.ernet.in/currsci/jul10/articles19.htm

This web review article contains detailed information and more than 160 references.

## Biosurfactants in environmental processes

http://gradworks.umi.com/35/50/3550595.html

This page links to a dissertation that describes the multiple environmental roles of microbial biosurfactants. Examples include their role in biodegradation and aid in swarming motility.

## New production processes for biosurfactants

http://www.igb.fraunhofer.de/en/competences/molecular-biotechnology/white-biotechnology/biosurf.html

This page describes an institutional programme to develop biosurfactants for industrial uses.

## Biosurfactants: Eugene Rosenberg

http://www.tau.ac.il/lifesci/departments/biotech/members/rosenberg/rosenberg.html

This personal web page contains links to many papers and patents pertaining to biosurfactants.

